# The Best Way to Deal Is with Cold Steel…Is It?—Ways of Dealing with Age and Ageing in Cardiac Surgery

**DOI:** 10.3390/jcm11237116

**Published:** 2022-11-30

**Authors:** Johannes M. Albes

**Affiliations:** Department of Cardiovascular Surgery, Heart Center Brandenburg, University Hospital Brandenburg Medical School, 16321 Bernau, Germany; johannes.albes@immanuelalbertinen.de

Everyone knows from their own experience what ageing means. All organ functions gradually decline, the body’s susceptibility to disease increases, and the frequency of the effects of ailments increases. Although we believe that ageing can be mitigated by good medical care and monitoring, as well as by a healthy lifestyle, proper nutrition, preventive measures, oral hygiene, etc., it is the time when ageing gradually turns into frailty and into the decline of neurocognitive functions, which start with forgetfulness and eventually develop into full-blown dementia. Of course, we have extended our youthful phase of life by about 10 years, but the reality is that there is no eternal youth, and certainly no fountain of youth. Each and every attempt to treat a disease in older patients is arduous and by no means a no-brainer in terms of success and sustainable restoration of the previous state.

Demographic change has indeed led to a steady increase in elderly and multimorbid patients. The percentage of patients aged 80 years or more has increased from 13.8% to 20.7% in the last 10 years according to the database of the German Society of Thoracic, Cardiac and Vascular Surgery (DGTHG) [1], a trend also observed in many western industrialized countries. Many of them suffer from cardiovascular diseases that require sound treatment strategies, i.e., reduction of surgical burden to reduce risks, low-invasive surgical procedures, and catheter-based interventions have already shown the desired results in terms of improved initial survival. However, many of these established strategies, such as transcatheter aortic valve implantation (TAVI), MitraClip, or percutaneous coronary intervention (PCI), come at a cost. Re-hospitalizations and inadequate procedural success may limit the medium- and long-term desired effects such as reduction of re-hospitalizations, preservation of mobility, and quality of life. While there is absolutely no dispute that age alone is a useful predictor of outcome, it is not at all clear how to assess the extent of frailty. Any physician will immediately recognize a frail person, but despite all the frailty scores currently in use, there is a lack of reliable parameters to provide a predictor for estimating outcome. Moreover, there is virtually no systematic treatment to reduce frailty, let alone interventions to address frailty.

Thus, five goals can be specified to deal with “ageing” in cardiac surgery:

1. Are there any novel therapeutic strategies, implants, or techniques to reduce the surgical burden in elderly and frail patients?
2. How can frailty be quantified and translated into a reliable predictor of outcome?
3. How can such predictors be used to stratify between surgical, interventional, or hybrid procedures for the best outcome for the patient?
4. Are there treatment options to reduce the extent of frailty before surgery or intervention?
5. Are there specific frailty management options to improve outcomes after surgical or interventional procedures?

None of these questions have comprehensively been answered yet, not even remotely. However, there are efforts underway, and some evidence has emerged.

1. New therapeutic strategies include, in particular, those that reduce surgical effort through a minimally invasive approach. Both mitral valve and aortic valve operations can be performed through small approaches that avoid a full sternotomy. This helps, but also creates new, previously unknown problems. Nonetheless, there is already solid evidence of good outcome and sustainability, though no breakthrough in terms of better outcome, only non-inferiority [2,3]. Unfortunately, CABG cannot be performed in a truly minimally invasive way. Many claim that OPCAB is minimally invasive because it avoids the extracorporeal circuit by performing bypass anastomoses on the beating heart instead. Over many years, a large number of papers were published, all of which were supposed to prove the superiority of OPCAB over CABG, but apart from some rather insignificant aspects, such as a shorter hospital stay or better performance in some subgroups, showed no real substantial advantage. Looking at the proportion of OPCAB versus CABG in the DGTHG database, there has been a steady increase in OPCAB numbers over the last 20 years, but still more than 75% of all bypass procedures are performed with the heart-lung machine. If OPCAB were as better as is often claimed, the proportion of CABG to OPCAB should actually have changed much more significantly [1,4]. The real minimally invasive approach, unequivocally accepted by all, is instead the transcatheter approach. When we look at the results of TAVI and MitraClip, it is clear that these strategies really avoid the surgical burden to the benefit of the patient [5,6,7]. As a result, these numbers are rising steeply, while the industry, recognising the enormous potential, has simultaneously developed several new or improved transcatheter valves and repair devices at a high rate. A mixed field in this regard is the treatment of the aorta with endovascular prostheses. In the ascending aorta and aortic arch, a hybrid approach combining transcatheter techniques with “mild” surgery, i.e., only peripheral vascular bypass manoeuvres flanking the implantation of the prosthesis and ensuring perfusion of the brain and spinal cord, is already a clinical reality. In this area, too, the industry has begun to meet the steadily increasing demand by developing ever more advanced endovascular prosthesis types. Indeed, these strategies show an advantage over conventional surgery in terms of early and late outcome and, in particular, a decrease in mortality [8]. However, they come at a price: an increase in re-interventions [9].
2. Quantifying frailty is an interesting phenomenon. As already stated, everyone recognises a frail patient at first sight, but it is difficult to put this into a score. However, we have made progress: there are different frailty scores, mainly related to muscle strength and mobility, but also to neurocognitive abilities, and some parameters actually serve as predictors. However, much is still elusive [10]. There is the old lady who comes through major heart surgery with a smile on her face, and then there is the supposedly spry silver-ager who develops systemic inflammatory syndrome (SIRS) postoperatively and despite all efforts does not climb out of the trench. It is completely unclear why some develop such postoperative SIRS and others do not [11]. So quantifying frailty has made some progress and predictors already exist, but ultimately the outcome for frail patients remains unpredictable.
3. This raises the question of how these predictors can help stratify for conventional surgery, hybrid or transcatheter procedures, or even avoid surgery or intervention altogether. Cardiac surgeons who have developed them and cardiologists who have adopted them rely on the EuroSCORE and STS scores to predict outcomes. This is helpful, but by no means sufficient. Time and again, it is seen that some patients do better, while others do worse. In this context, it should be borne in mind that many rare diseases, such as cirrhosis of the liver [12], do not even appear in these scores, as a statistical evaluation of their impact is not possible when using the underlying database, because these diseases simply do not occur in sufficient numbers for such a statistical evaluation.
4. A new approach aims to improve the patient’s condition before major heart surgery. It may well be called “tuning” or “doping”. Several organ functions can actually be optimised to a certain degree—not much, but enough to have the few percent more resilience needed to get through the difficult early perioperative period. While kidney function and heart function can be improved to some degree, other functions cannot. Liver function eludes improving measures. Good results can be achieved by optimising another, often overlooked organ: the blood, e.g., by reducing preoperative anaemia with the help of erythropoietin [13] or preoperative albumin substitution [14]. Some recent efforts are aimed at improving neurocognitive function and thus reducing susceptibility to the development of severe postoperative delirium, which is known to increase not only morbidity by deep sternal wound infection [15] but also mortality. However, there is no breakthrough in sight. Nutrition may play a role. It is known that either the obese as well as the cachectic patients do have a poorer outcome than the normal or slightly overweight patients. Whether or not this can be corrected in a short period prior to surgery appears to be questionable. Perhaps, this is something to be dealt with over a long period and is thus rather a lifestyle issue [16]. A brand-new multicentre trial is currently underway in which elective patients are enrolled in rehabilitation before surgery to get them ready for surgery [17]. We will wait and see what happens.
5. The management of frailty in the perioperative setting points to organ-preserving strategies, but also to the avoidance or at least reduction of postoperative delirium. In addition, mobilisation is key, especially in the elderly, to improving ventilation and thus reducing the risk of developing pneumonia. The maintenance of the gut microbiome, especially in frail patients, is not sufficiently considered, although recent evidence shows that a well-functioning microbiome has a variety of beneficial effects not only on organ function [18] but also on the psyche by the so-called brain–gut axis [19].

Is there a therapeutic end somewhere in old age? From a biological point of view, we are asymptotically approaching a limit, even if, mathematically speaking, we will never reach it. At present, we can say that patients over 80 have become the norm. The same could be true in about 10 years for patients over 90. A therapeutic end can only be arbitrarily brought about by health policy. It may well be that at some point health systems can no longer afford to provide every patient with expensive treatment methods and set age limits for reasons of fairness towards younger patients in need of treatment. From an ethical point of view, however, this is questionable, but eventually some limits will be introduced anyway. Perhaps not on the basis of set age limits, but rather with the help of quality monitoring tools. By setting strict thresholds for certain procedural and outcome variables, high-risk patients, such as elderly patients, will eventually be denied treatment in order to stay within the set limits and thus avoid sanctions. In many countries, such a system has already been introduced and the responsible bodies are slowly but steadily tightening the reins. This is cynical, but those who provide the money for health care are in a position to do so [20].

Nevertheless, it is time to consider old age and frailty not as such, but as a condition that can be assessed with reasonable accuracy and that can also be treated preoperatively or postoperatively or postinterventionally to improve the patient’s condition (Figure 1). The ultimate goal for the patients is easily defined: living at home, being mobile, requiring little nursing assistance, and having a good quality of life without requiring re-hospitalizations. The time for shrugging shoulders in the treatment of ageing patients is over; the time is ripe for clear strategies to define, assess, stratify, and treat the ageing patient. Some things have already been recognised, but much is still up in the air.

## Figures and Tables

**Figure 1:**
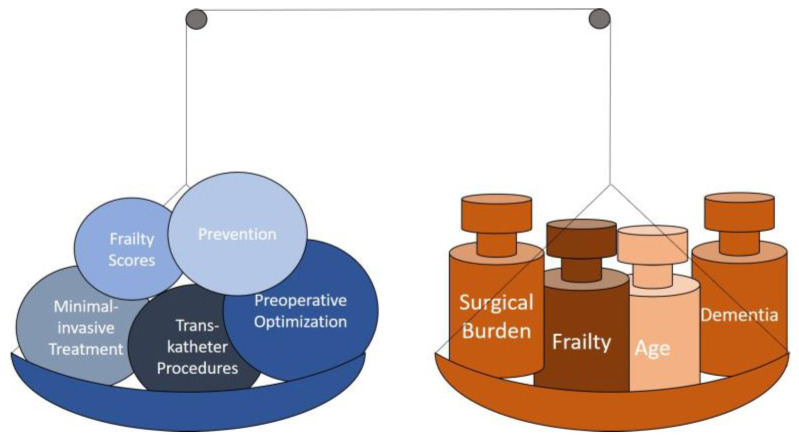
How to reduce the burden.
